# Implementing buprenorphine in addiction treatment: payer and provider perspectives in Ohio

**DOI:** 10.1186/s13011-015-0009-2

**Published:** 2015-03-28

**Authors:** Todd Molfenter, Carol Sherbeck, Mark Zehner, Andy Quanbeck, Dennis McCarty, Jee-Seon Kim, Sandy Starr

**Affiliations:** University of Wisconsin–Madison, 1513 University Avenue, Madison, Wisconsin 53706 USA; Oregon Health & Science University, 3181 S.W. Sam Jackson Park Rd., Portland, Oregon 97239-3098 USA; University of Wisconsin–Madison, School of Education, 1057 Educational Sciences, Madison, WI 53706 USA; Ohio Department of Mental Health and Addiction Services (OhioMHAS), 30 East Broad Street, 8th Floor, Columbus, Ohio 43215 USA

**Keywords:** Payer perspectives, Buprenorphine, Opioid addiction, Qualitative research, Medication-assisted treatment

## Abstract

**Background:**

Buprenorphine is under-utilized in treating opioid addiction. Payers and providers both have substantial influence over the adoption and use of this medication to enhance recovery. Their views could provide insights into the barriers and facilitators in buprenorphine adoption.

**Methods:**

We conducted individual interviews with 18 Ohio county Alcohol, Drug Addiction, and Mental Health Services (ADAMHS) Boards (payers) and 36 addiction treatment centers (providers) to examine barriers and facilitators to buprenorphine use. Transcripts were reviewed, coded, and qualitatively analyzed. First, we examined reasons that county boards supported buprenorphine use. A second analysis compared county boards and addiction treatment providers on perceived barriers and facilitators to buprenorphine use. The final analysis compared county boards with low and high use of buprenorphine to determine how facilitators and barriers differed between those settings.

**Results:**

County boards (payers) promoted buprenorphine use to improve clinical care, reduce opioid overdose deaths, and prepare providers for participation in integrated models of health care delivery with primary care clinics and hospitals. Providers and payers shared many of the same perceptions of facilitators and barriers to buprenorphine use. Common facilitators identified were knowledge of buprenorphine benefits, funds allocated to purchase buprenorphine, and support from the criminal justice system. Common barriers were negative attitudes toward use of agonist pharmacotherapy, payment environment, and physician prescribing capacity. County boards with low buprenorphine use rates cited negative attitudes toward use of agonist medication as a primary barrier. County boards with high rates of buprenorphine use dedicated funds to purchase buprenorphine in spite of concerns about limited physician prescribing capacity.

**Conclusions:**

This qualitative analysis found that attitudes toward use of medication and medication funding environment play important roles in an organization’s decision to begin buprenorphine use and that physician availability influences an organization’s ability to expand buprenorphine use over time.

Additional education, reimbursement support, and policy changes are needed to support buprenorphine adoption and use, along with a greater understanding of the roles payers, providers, and regulators play in the adoption of targeted practices.

## Background

### Buprenorphine adoption

Buprenorphine is a pharmacotherapy that acts as a partial μ-opiate-receptor agonist [1]. When combined with naloxone, a full μ-opiate-receptor antagonist that can counter the effects of opioids, there is diminished potential for buprenorphine to be abused [2]. Agonist pharmacotherapy for opioid dependence (i.e., buprenorphine, buprenorphine/naloxone combinations, and methadone) improves retention in addiction treatment and reduces the use of illicit opioids [3,4]. Until 2002, methadone, a full μ-opiate-receptor agonist, was the primary pharmacotherapy for opioid dependence; however, methadone is only available in regulated settings that supply limited take-home medication, and is prone to causing adverse reactions [5]. Within this environment, buprenorphine and buprenorphine/naloxone combinations were projected to play an important role in the treatment of opioid addiction when approved by the Food and Drug Administration (FDA) in 2002 for use by authorized physicians [6]. Adoption, however, has been slow, especially among publicly-funded addiction treatment centers; only 17% percent of specialty treatment centers who accept public funds and provide outpatient, intensive outpatient, or residential addiction services offer buprenorphine treatment [7].

### Payer role

Payers and regulators, in their oversight role, influence the structure of treatment systems and the practices used in treatment centers. Knudsen and Abraham [8] found that treatment programs were more likely to have adopted pharmacotherapy for addiction, including but not limited to buprenorphine, if they perceived greater support for medications by the Single State Authority overseeing publicly-funded substance abuse treatment services. Treatment programs in states that provided public insurance coverage for buprenorphine were more likely to provide the medication [9]. The payers’ role is not limited to reimbursement policy; payers also influence regulatory policy, contracting requirements, and provider education [10]. Additionally, payers’ level of buprenorphine support varies, and few payers apply the same policy rules to buprenorphine therapy as to they do to medication therapies common to the treatment of other chronic disease conditions [11]. Despite payers’ instrumental role and mixed support, their perceptions of facilitators and barriers to buprenorphine use have been understudied in the health services literature [8,9,12].

### Provider facilitators and barriers

Payers contract with treatment agencies (providers) to provide treatment services, including buprenorphine treatment. Addiction treatment centers using buprenorphine are more likely to be accredited or licensed [11], participate in managed care arrangements [11], provide detoxification services [9,13,14], employ physicians [13,15], and offer other medication-oriented treatments [14,16,17]. A lack of reimbursement for buprenorphine [18], philosophical resistance to medication therapy [19], and limited availability of buprenorphine prescribers [7] inhibit use of buprenorphine in specialty treatment settings. Treatment practitioners’ perceptions of facilitators and barriers to buprenorphine use and payer priorities must inform policy if buprenorphine-assisted treatment is going to reduce opioid addiction and overdose deaths.

### Study setting: opioid trends in Ohio

Nationwide, non-medical use of opioids is significantly influencing public health [20]. Ohio has the 12^th^ highest drug overdose mortality rate in the United States, and Ohio has implemented many of the same opioid prescription misuse policies tested in other states [21]. In 2011, 66.7 doses of prescription opioids were purchased per Ohio citizen, and 1.2 Ohioans died per day due to unintentional overdose of prescription opioids [22]. The increase in patients seeking treatment for opioid dependence challenges Ohio’s publicly-funded system of specialty addiction treatment providers. Ohio treatment providers report that opioid-dependent patients make up more than a quarter of all clients served, and nearly 80 percent of providers reported increased wait times for assessment services due to increased treatment demand [23].

Ohio currently (2015) provides outpatient methadone therapy, but is limited to 13 locations. With the small number of methadone locations, methadone’s limited take-home availability, and the prevalence of opioid abuse, buprenorphine treatment is seen as a key strategy for reducing the adverse effects of opioid dependence in Ohio [24]. At present, 22% of Ohio treatment providers use buprenorphine to support recovery and 43% make referrals to physicians authorized to prescribed buprenorphine [23]. This is higher than the national buprenorphine use rate of 17% [7], but falls considerably short of 100% use.

Publicly funded addiction treatment in Ohio is administered through Alcohol, Drug Addiction, and Mental Health Services (ADAMHS) boards (henceforth referred to as “county boards”) (n = 53) and each board represents one to five counties. ADAMHS boards support specialty addiction treatment services using state and local appropriations combined with the federal Substance Abuse and Prevention Treatment (SAPT) block grant. The Substance Abuse and Mental Health Services Administration (SAMHSA) provides the SAPT funds, which are intended for the uninsured and fund up to 55% of addiction treatment services [25]. Within states, counties and other fiscal intermediaries often perform logical and meaningful roles in the distribution of addiction funds. In the United States, 34% of these addiction treatment funds are allocated by county or regional entities.

The county board structure creates many contrasting payer environments in Ohio. Our qualitative study compares perceived facilitators and barriers to buprenorphine adoption among Ohio’s payers (the county boards) and providers, and describes why payers and providers do or do not support the use of buprenorphine to treat opioid dependence.

## Methods

### Setting and recruitment

A community trial studied an intervention to increase use of buprenorphine and other medications for opioid dependence in Ohio. County boards and substance abuse treatment provider interviews were conducted during the pre-implementation phase of this trial [26]. County board participants (N=18) and treatment provider agency participants (N=36) with greater than 100 admissions per year were recruited (September 1, 2012 to October 31, 2012) and completed the individual interviews (November 1, 2012 to February 28, 2013) by telephone. The county boards represented 27 of Ohio’s 88 counties, and these boards support 65 treatment agencies with more than 100 annual admissions. A representative sample of county board participants was selected, stratified on 20% opioid admissions of total admissions (above/below) and 200,000 people covered (above/below). Three county boards that were not participating in the trial were interviewed to reduce selection bias. No other Ohio county boards were approached to participate in this study. All county boards referred two organizations. These organizations represented 55.4% (36/65) of the organizations eligible in those county board areas. Participating counties represented 44.2% (65/315) of all treatment providers in Ohio with more than 100 admissions per year. No organizations refused to participate in the qualitative interviews.

For the county board interviews, the board’s highest-ranking clinical officer or the executive director completed the interview. These individuals tend to be responsible for adopting new clinical practices; they also encounter the facilitators and barriers directly during clinical practice implementation. Two treatment agencies were interviewed in each county board area to capture multiple perspectives. A clinical manager with direct exposure to the facilitators and barriers of the buprenorphine adoption process completed the agency interviews.

### Data collection procedures

Three members of the research team conducted interviews. The principal investigator trained two members of the research team and observed their first two county board and provider interviews. The interview guide was pilot tested with two county boards and two providers. The final interview guide addressed knowledge of barriers and facilitators to buprenorphine use for the county boards and providers. The county boards were asked whether or not they support buprenorphine use, and what caused them to support or not support buprenorphine adoption. These were asked as general questions, and no specific issues were solicited. The semi-structured format allowed for standardization across interviews and for the interviewer to probe for additional information when appropriate.

The research team used data from the state’s pharmacy management and addiction treatment database to calculate buprenorphine use rates by county (i.e., individuals with a buprenorphine prescription during 2012 divided by the number of opioid dependent individuals admitted to the public treatment system by county). The study received approval from the institutional review boards at the University of Wisconsin–Madison and the Ohio Department of Health.

### Data analysis

The review of the qualitative data followed a summative qualitative content analysis procedure [27]. Interviews were transcribed for review and four members of the research team coded the transcripts. The multi-disciplinary research team (with backgrounds in addictions, operations management, and education) ensured multiple viewpoints and vigorous discussion of the data. The first step in the coding process involved open coding. Transcripts were coded and analyzed using ATLAS.ti [28]. Two team members coded each transcript independently to enhance reliability. An inductive approach for analyzing qualitative data identified codes and concepts within each interview [29]. Codes and concepts were grouped by county board and provider. The researchers sought consensus on code nomenclature and content to ensure more consistent coding. Upon reaching consensus on broad thematic codes and their working definitions, the team revisited the interviews with the preliminary coding scheme. At this stage, to ensure consistency across coders, we examined agreement across a random selection of interviews. Items with discrepant codes were discussed until consensus was reached. No coded comments were discarded throughout the coding process. When coding was complete, queries of specific codes were generated. Summaries were generated for the common themes, with examples of text from each theme. Quotes were chosen to illustrate themes expressed by respondents based on clarity, brevity, variety, and their representation of similar comments from a range of participants.

## Results

### Sample characteristics

Listed in Table 1 are the characteristics of the individuals surveyed, the county boards, and provider agencies.

**Table 1:** **Participant site characteristics – county boards and provider agencies**

**Interviewee site characteristics**		**N**	**%**
**County (n = 18)**			
**County board**	**Title**	**Count**	**%**
General job description	Executive Director	8	44.4
	Vice President/Director	9	50.0
	Medical Director	1	5.6
People served	<1000	4	22.2
	1000-1999	6	33.3
	2000-3999	5	27.8
	4000+	3	16.7
Funds available for buprenorphine*	Medicaid	18	100.0
	SAPT Block Grant	7	38.9
	Tax Levy	13	72.2
	State Grant	1	7.7
	Federal Grant	3	16.7
**Provider (n = 36)**			
General job description	President/CEO/Executive Director	15	41.6
	Vice President/Director	17	50.0
	Medical Director	4	11.1
Services*	Detoxification	12	33.3
	Outpatient	30	83.3
	Intensive Outpatient	26	72.2
	Residential	17	47.2
Physicians on staff	Yes	19	52.8
	No	17	47.2
Full-time staff equivalents (FTEs)	0-50	17	47.2
	51-100	12	33.3
	100+	7	19.4
Use of methadone on-site	Yes	5	13.9
	No	31	86.1
Buprenorphine use	Prescribe	15	41.7
	Refer to other providers	13	36.1
	No use	8	22.2

*Multiple responses were possible.

#### County board reasons for buprenorphine adoption

All county boards interviewed stated support for the use of buprenorphine for opioid treatment, with no boards opposed to use of buprenorphine. County boards reported three reasons for supporting the adoption of buprenorphine: the escalating rates of opioid dependence/opioid crisis, the need for better care, and integration with general health care.

**Responding to the opioid crisis:** County boards saw a need to address opioid misuse in their counties because of the increased demand for detoxification and care. As one board representative stated:

*“We identified this huge increase in opioid addiction coming through the door. That is what kicked off our search for what are the best practices out there and that is how we got involved with buprenorphine and medication-assisted treatment.” (Board Quote)*

**Better clinical care:** County board respondents expressed a desire for better clinical care for opioid misuse (i.e., care that was individualized, had better retention rates, and appealed to younger adults).

*“We’re really looking at buprenorphine to get people retained in treatment until they have the skills to stay sober in the long term, until they have the recovery supports around them.” (Board)*

**Preparing for integration:** County boards anticipated that addiction treatment organizations would need to offer addiction medication therapy to be part of integrated care structures where behavioral health (e.g. addiction treatment and mental health) and general medical (e.g. primary and hospital care) professional services are coordinated and delivered simultaneously.

*“Now is the time to be smart about everything going into the integration of behavioral health and medical health. It’s like the train is running and it won’t be too long before it starts to move and it seems obvious for medication-assisted therapy to have its body inside one of the train cars.” (Board)*

### County board and provider barriers and facilitators

Within a receptive environment for buprenorphine adoption and use, boards and providers identified potential barriers and facilitators to buprenorphine.

#### County board and provider barriers

Barriers among the county boards and providers were aggregated into five thematic areas: a) negative attitudes toward the use of agonist pharmacotherapy, b) lack of awareness/understanding, c) limited physician availability, d) insufficient funds, and e) diversion concerns.

Most of the themes and sub-themes were consistent between the county board and provider respondents, with few noticeable exceptions. Providers were more concerned about addiction treatment providers’ lack of knowledge of buprenorphine, and also about recovery groups’ hostility toward use of medications for addiction treatment. Representative quotes illustrate the barrier themes and sub-themes, with whether the quote came from a board or provider interview noted.

### A) Negative attitudes toward use of medication

**Providers:** Staff orientation toward abstinence-based approaches was often the stated cause of resistance to medication therapy.

*“We have had a pretty big struggle in our community, philosophically, about some of our providers…that truly believe that medication-assisted treatment is substituting one drug for another.” (Board)*

*“I got some staff that are questioning why we would give a drug to an addict.” (Provider)*

**Recovery groups:** Negative thinking about medication can extend to recovery groups. Providers have found this to be a challenge when seeking recovery supports for patients on pharmacotherapies.

*“I think the Alcoholics Anonymous (AA) and Narcotics Anonymous (NA) community has a lot of prejudice against opioid maintenance therapy.” (Provider)*

*“Those in recovery say if you are taking a pill, then you’re not in recovery.” (Board)*

### B) Lack of awareness/understanding

**Addiction provider not knowledgable about buprenorphine:** A sub-theme among providers was that buprenorphine would be more readily accepted with more training and a better understanding of buprenorphine.

*“I think our staff would like to have more education beyond just the initial, here is the chemical make-up of buprenorphine, and here is how it works.” (Provider)*

**Physicians not knowledgable about buprenorphine:** Physicians not specializing in addiction treatment were perceived to be possibly administering buprenorphine without adequate addiction and behavioral health therapy services.

*“Most physicians use it [buprenorphine] as they do other pharmacological aids in the sense, that it is a pill, rather than wrapping around a holistic approach with patients.” (Board)*

### C) Limited physician availability

**Physician unwillingness to prescribe buprenorphine:** Physicians reportedly did not want to begin prescribing buprenorphine because of concerns of working with addiction clientele.

*“Doctors do not want to deal with this population.” (Board)*

*“We went around to identify different physicians that would be willing to be involved as a referral site for us and we got a tremendous amount of negative or uninterested response.” (Provider)*

**Limited physician prescribing capacity:** Capacity was constrained by government policy (DATA 2000) [30] allowing physicians to only treat up to 30 patients at a time during the first year of certification and 100 patients thereafter.

*“We are stuck with federal regulation that says you can only have 100 patients per prescriber.” (Provider)*

*“This isn’t unique to [our] county… I’ve run into this in other counties and even statewide… physicians get involved, and they get to their 30-patient cap or if they’re beyond their first year, and they get to their 100-person cap. And then they don’t know how to discontinue them, and they’re stuck with this immovable object of a caseload until people start to attrite or they discontinue.” (Board)*

### D) Insufficient funds

**Insufficient funds for addiction treatment:** The county boards and providers expressed frustration and concern with the inability to pay for addiction treatment (with or without buprenorphine) for uninsured patients. However, when SAPT block grant funds were the only option for payment for buprenorphine, county boards and providers expressed a need to preserve existing therapy-based services instead of reallocating funds for buprenorphine use.

*“Well again it’s the lack of funding, we really don’t have any money to fund medication-assisted therapy.” (Board)*

*“We were struggling with funding issues of how it would be paid for so medications could at least be cost neutral.” (Provider)*

### E) Diversion concerns

**Diversion Concerns:** Concerns about patients selling their buprenorphine can be a disincentive for organizations to adopt buprenorphine. These concerns also challenge existing efforts for those who have adopted buprenorphine.

*“I think that people outside of the clinical community have this understanding now that buprenorphine has a street value; the whole issue with diversion. I think it is what we face the most concerning the medication.” (Provider)*

#### County board and provider facilitators

The facilitators were aggregated into three thematic areas related to a) providers are knowledgeable (about buprenorphine); b) criminal justice involvement; and c) funding. Again, the county boards and providers tended to share the same perceptions of facilitators. Providers expressed a much greater appreciation for having access to physicians who supported buprenorphine use, while county boards perceived the criminal justice system had a stronger influence on addiction treatment practices.

### A) Provider knowledge about buprenorphine

**Addiction providers’ knowledge about buprenorphine:** County boards and providers frequently cited high levels of provider knowledge as assisting with buprenorphine adoption.

*“Every single one of our counselors and therapists is well aware and on board with medication as a fundamental component of treating certain addictions.” (Provider)*

**Specialty physician knowledge about buprenorphine:** In this provider-dominant theme, physicians dedicated to addiction care were perceived to be knowledgable about this treatment option.

*“I think in terms of the community, those that are involved with the alcohol and treatment system are very knowledgable.” (Provider)*

### B) Criminal justice involvement

**Community stakeholder involvement – criminal justice:** This predominately county board theme expressed an appreciation for the criminal justice system’s influence on addiction treatment practice and policy. The county boards noted how criminal justice system referrals to addiction treatment programs could facilitate buprenorphine practice.

*“It is most important to have criminal justice system behind use of buprenorphine. They are just huge in our county.” (Board)*

### C) Funding available

**County funding for buprenorphine:** The county boards were seen as one of several entities that provided financial support for buprenorphine use. The counties’ ability to allocate tax levy funds (separate from SAPT block grant funds) to support use of buprenorphine for uninsured consumers was noted as an important funding source.

*“Seventy-five per cent of our funding for buprenorphine is through our local levy. We could not provide buprenorphine therapy without these funds.” (Board)*

### Use of buprenorphine

The last analysis was to discern what themes were present in county boards that had low or high utilization of buprenorphine. The county boards had varying buprenorphine use rates in 2012, with opioid admissions ranging from 4% to 47% with a mean of 20% (SD 12%). Four county boards with buprenorphine use rates for opioid admissions below 10% were compared with four boards that had rates 30% or greater.

Boards with low buprenorphine use rates shared concerns about negative attitudes toward medication. They reported that negative attitudes towards buprenorphine hamper its uptake in the provider community.

*“Our providers have considerable resistance to medication-assisted therapy because it does not follow 12-steps traditions.” (Board)*

Boards with high buprenorphine use rates dedicated funds to purchase buprenorphine, but reported limited physician availability as a principal barrier.

*“We have funds dedicated to buprenorphine therapy, but cannot always expend them due to a shortage of physician prescribers.” (Board)*

## Discussion

The analysis addresses how the increase of opioid misuse affects the county boards and providers in Ohio. The county boards uniformly expressed concerns about the growing use of prescription and non-prescription opioids and perceived buprenorphine therapy as a viable treatment option. Statewide, the county boards in Ohio prioritize opioid misuse and have policies to address it [31]. The county boards’ motivation to encourage buprenorphine use was based on their fundamental desire for better clinical care for opioid dependent patients.

It is not uncommon for county boards (or other payers) and providers to have conflicting opinions on issues related to payment policy, clinical care, quality metrics, and necessary treatment capacity [32,33]. However, in this qualitative assessment, county boards and providers had similar thoughts on the barriers and facilitators to buprenorphine use. County boards and treatment providers may be aligned on this issue because they influence each other’s perceptions and must collaborate on a response to the opioid crisis and its effect on the addiction treatment system and local communities.

### Review of the findings: based on three thematic drivers

#### Ideology/Knowledge

Ideology precluding use of medication and lack of knowledge of the benefits of medication were identified by study participants as important issues. Long-standing traditions and firmly held beliefs steeped in 12-step tradition have been found to influence counselor attitudes towards use of buprenorphine and other medication-assisted therapies [34]. Yet, the abstinence-only approach to detoxification and treatment for opioid disorders has not been effective [35]. On the other hand, medication-assisted therapy possesses an evidence base that supports greater treatment retention and reduced use of illicit opioids [4,36]. Hence, those preferring the behavioral approaches over medication-assisted treatment may be placing ideology over the evidence-base.

Participants’ concerns about anti-medication ideology extended to physicians as well as to counselors. They felt that buprenorphine-specific training could help change attitudes in these key clinical roles.

#### Physicians

Studies related to physician’s willingness to prescribe buprenorphine tend to be based on special circumstances such as HIV care [37] or focused on primary care settings [38,39]. Other authors have recommended that more attention be given to physicians working within addiction treatment programs [40].

**Figure 1:**
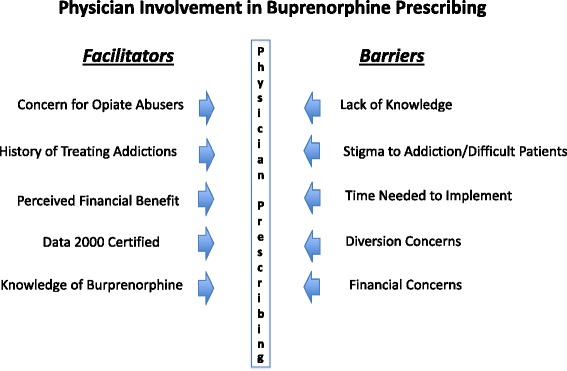
**Physician involvement in buprenorphine prescribing.**

As this analysis explored facilitators and barriers to buprenorphine use in addiction treatment programs, it found a complex set of facilitators and barriers to physician involvement in buprenorphine prescribing (Figure 1). Barriers began with physicians’ aversion to addiction care. Respondents mentioned physician concerns about diversion and insufficient time to adopt buprenorphine prescribing and monitoring into their practice as additional forces influencing physician adoption. Facilitating forces, conversely, are based on physicians being drawn to addiction care.

#### Funding

Medicaid reimbursement has been associated with greater use of addiction medications [9,25]. In Ohio, Medicaid pays for buprenorphine therapy, but a significant number of patients remain without health insurance to pay for buprenorphine medication and associated ancillary services. This study did confirm the importance of a funding source for buprenorphine therapy and discovered another payment source for these medications: use of county tax levy funds.

### Implications

The analysis of high and low buprenorphine use rates suggests that county funding is a primary driver of buprenorphine use in Ohio. Accordingly, payers wanting to expand use of buprenorphine should assure that payment is available for the buprenorphine medication, as well as associated physician time, laboratory tests, and counseling. Our analysis, however, suggests that county funding is necessary but not sufficient. The implementation science literature draws a distinction between *adoption* and *implementation* of a practice, with adoption being the first time a person or entity applies the practice and implementation representing the frequency of application after adoption [41].

For adoption of buprenorphine in Ohio, it seems that funding and mindsets are important. The high-use buprenorphine county board areas had funding for buprenorphine; in low-use buprenorphine county boards areas, oppositional ideologies created barriers to buprenorphine implementation. Yet, two of the low-use county boards had county levy dollars available to support use of buprenorphine. Hence, a combination of amenable ideology and funding supports may be needed to create an attractive environment for adoption activities. Buprenorphine-specific training for counselors and physicians can begin to increase knowledge, resulting in counselors who are more likely to find buprenorphine as acceptable and effective for treating opioid dependence [42,43].

Physician capacity issues were consistently raised as an issue in increasing use following adoption. The DATA 2000 cap of 30 patients per physician in year one and 100 thereafter [30] creates a clear structural barrier for physicians working in addiction treatment settings. This is particularly the case for physicians who have caseloads of patients on long-term buprenorphine therapy occupying their existing slots. New physicians must be recruited once the existing buprenorphine prescribers are treating their allotted number of patients. This can be difficult when programs must recruit physicians who do not have a background in addiction treatment and lends credence to the proposition by Wood, et al. [44] to provide greater physician education in addiction medicine. All three of the high volume buprenorphine county board areas needed additional physician buprenorphine prescribing capacity, underscoring the importance of this factor in promoting additional buprenorphine use.

### Study limitations

There are limitations based on the sample. Potential selection bias may exist in the county board sample because 15 of the 18 county boards that participated in the qualitative interviews agreed to participate in an intervention study on buprenorphine adoption. The sample only represents the public treatment system in Ohio. However, Ohio has been considered to be on the forefront of states seeking to remedy non-medical use of prescription opioids [45].

Another limitation is that many beliefs and attitudes were attributed to physicians in the analysis. These beliefs and attitudes were based on payers’ and providers’ observation from regular interactions with physicians, yet were not directly provided by practicing physicians. Additional analyses of buprenorphine beliefs and attitudes should include physicians in the sample to determine how their perspectives compare to those of payers and providers.

Lastly, the interviews were conducted with a limited group of key decision makers from the county boards and provider organizations. While we assumed their comments were representative of issues occurring in the environment, a greater set of interviews within the organizations surveyed as well as other organizations could provide additional input for consideration.

## Conclusions

Our study was specific to Ohio, but barriers and facilitators are not unique to this state. This research suggests initial adoption of buprenorphine requires funding and favorable mindsets, while expanded use requires adequate physician capacity. Education can increase knowledge and reduce prejudices against medication-assisted treatment, and policy can play a role in funding supports and physician capacity. Ongoing efforts to promote buprenorphine reimbursement and to remove the physician prescribing caps could help expand use. Lastly, continuing to develop a greater understanding of the unique roles of payers, providers, and regulatory bodies will promote adoption and implementation of buprenorphine and other evidence-based practices.
